# TRPV4 inhibition prevents increased water diffusion and blood-retina barrier breakdown in the retina of streptozotocin-induced diabetic mice

**DOI:** 10.1371/journal.pone.0212158

**Published:** 2019-05-02

**Authors:** Maricruz Orduña Ríos, Ramsés Noguez Imm, Nicole Marilú Hernández Godínez, Ana María Bautista Cortes, Dayana Deyanira López Escalante, Wolfgang Liedtke, Atáulfo Martínez Torres, Luis Concha, Stéphanie Thébault

**Affiliations:** 1 Instituto de Neurobiología, Universidad Nacional Autónoma de México (UNAM), Campus UNAM-Juriquilla, Querétaro, México; 2 Department of Medicine and Neurobiology, Center for Translational Neuroscience, Duke University Medical Center, Durham, North Carolina, United States of America; Children’s Hospital Boston, UNITED STATES

## Abstract

A better understanding of the molecular and cellular mechanisms involved in retinal hydro-mineral homeostasis imbalance during diabetic macular edema (DME) is needed to gain insights into retinal (patho-)physiology that will help elaborate innovative therapies with lower health care costs. Transient receptor potential cation channel subfamily vanilloid member 4 (TRPV4) plays an intricate role in homeostatic processes that needs to be deciphered in normal and diabetic retina. Based on previous findings showing that TRPV4 antagonists resolve blood-retina barrier (BRB) breakdown in diabetic rats, we evaluated whether TRPV4 channel inhibition prevents and reverts retinal edema in streptozotocin(STZ)-induced diabetic mice. We assessed retinal edema using common metrics, including retinal morphology/thickness (histology) and BRB integrity (albumin-associated tracer), and also by quantifying water mobility through apparent diffusion coefficient (ADC) measures. ADC was measured by diffusion-weighted magnetic resonance imaging (DW-MRI), acquired *ex vivo* at 4 weeks after STZ injection in diabetes and control groups. DWI images were also used to assess retinal thickness. TRPV4 was genetically ablated or pharmacologically inhibited as follows: left eyes were used as vehicle control and right eyes were intravitreally injected with TRPV4-selective antagonist GSK2193874, 24 h before the end of the 4 weeks of diabetes. Histological data show that retinal thickness was similar in nondiabetic and diabetic wt groups but increased in diabetic *Trpv4*^-/-^ mice. In contrast, DWI shows retinal thinning in diabetic wt mice that was absent in diabetic *Trpv4*^-/-^ mice. Disorganized outer nuclear layer was observed in diabetic wt but not in diabetic *Trpv4*^-/-^ retinas. We further demonstrate increased water diffusion, increased distances between photoreceptor nuclei, reduced nuclear area in all nuclear layers, and BRB hyperpermeability, in diabetic wt mice, effects that were absent in diabetic *Trpv4*^-/-^ mice. Retinas of diabetic mice treated with PBS showed increased water diffusion that was not normalized by GSK2193874. ADC maps in nondiabetic *Trpv4*^-/-^ mouse retinas showed restricted diffusion. Our data provide evidence that water diffusion is increased in diabetic mouse retinas and that TRPV4 function contributes to retinal hydro-mineral homeostasis and structure under control conditions, and to the development of BRB breakdown and increased water diffusion in the retina under diabetes conditions. A single intravitreous injection of TRPV4 antagonist is however not sufficient to revert these alterations in diabetic mouse retinas.

## Introduction

If clinical treatments exist for diabetic macular edema (DME), a complication of diabetes that results from an imbalance between retinal fluid entry and fluid exit, leading to intraretinal and subretinal fluid accumulation in the macula region of the retina [1], offering therapeutic alternatives is still a challenge. Under normal conditions, the inner blood-retina barrier (BRB), formed by the tight junctions between the intraretinal vascular endothelial cells, limits fluid entry. Dynamic interactions with pericytes, astrocytes, Müller glia, and microglia also contribute to the inner BRB function. Additionally, retinal fluid entry is limited by the retinal pigment epithelium junctions and the outer limiting membrane (OLM), which both form the outer BRB. Fluid exit is ensured by Müller glia and retinal pigment epithelium that continuously drain water and osmolytes from the retina.

During DME, permeability through retinal barriers increases, causing protein leakage within the interstitial retinal tissue that will be accompanied by water accumulation [2]. This vasogenic edema may be accompanied by an increase in intracellular fluid volume (cell swelling) [3, 4]. Additionally, decreased drainage functions account for decreased fluid exit in DME. Cell loss, OLM disruption, and deregulation of transporters and ion/water channels contribute to BRB breakdown and reduced drainage in DME. These pathological mechanisms are a consequence of fundamental pathways activated by chronic hyperglycemia, including inflammatory ones [1, 5, 6]. Recent evidence supports the view that transient receptor potential cation channel subfamily vanilloid member 4 (TRPV4) participates in BRB permeability and drainage functions of the retina.

Functionally coupled to aquaporin-4, TRPV4 is necessary for the regulatory volume decrease under hypo-osmotic conditions in retinal Müller glia endfeet [7]. Furthermore, *Trpv4*^-/-^ mice show disrupted OLM [8]. TRPV4 has also been implicated in the regulation of barrier permeability, but this is subject of debate. While studies showed that TRPV4 activation promotes barrier resistance [9, 10], others reported that its stimulation provokes barrier breakdown [11–19]. An additional confounding aspect is that diabetes or hyperglucemic-mimicking conditions were associated with TRPV4 down-regulation in different cell types, including rat retinal microvascular endothelium [20], mesenteric artery endothelial cells [21], and human collecting duct cells [22]. Based on inconsistent observations in *Trpv4*^-/-^ mice, it has been suggested that the intricate role of TRPV4 may relate to its presence in different vascular beds and most cellular components of the neurogliovascular unit [23–25]. In the retina, TRPV4 is expressed in neurons, Müller glia, astrocytes, endothelial cells, and retinal pigment epithelium [8, 20, 26, 27].

Reduced TRPV4 expression may be a compensatory effect in diabetes. Indeed, TRPV4 activation mediates and amplifies inflammatory responses [15, 28–34], including edema [15, 34]. TRPV4 inhibitors resolve edema in several organs [35–40] and TRPV4 selective antagonists (RN-1734 and GSK2193874) mitigate BRB breakdown in diabetic rats [8]. The role of TRPV4 in retinal hydro-mineral homeostasis should therefore be better characterized under control and diabetic conditions.

Although none of the existing experimental models recapitulate DME, the commonly used animal model of streptozotocin-induced diabetic retinopathy shares similarities that provide insight into the genesis and evolution of DME. In addition to a rapid onset of hyperglycemia, this model displays signs of early diabetic retinopathy, including blood-retina barrier (BRB) breakdown [41]. Mice have been used less frequently than rats as models in studies of DME, but they exhibit features of diabetic retinopathy [42, 43]. Also, the mouse genome can be relatively easily manipulated to inhibit a channel, which is a major advantage over pharmacological tools. In this study, we first analyzed retinas of *Trpv4*^-/-^ mice subjected to 4 weeks of experimental diabetes induced by streptozotocin. Retinal edema was assessed by measuring morphology/thickness, BRB integrity, and water diffusion using histology, an albumin-associated tracer, and diffusion-weighted magnetic resonance imaging (DW-MRI), respectively. DW-MRI is a MRI modality that can measure apparent diffusion coefficient (ADC), a sensitive biomarker of water mobility [44], whose increase is observed in models of vasogenic edema [45]. A previous study showed diffuse retinal edema in diabetic rats using *in vivo* DWI [46]. MRI analysis showed that diabetic male C57BL/6 mouse retinas are not thicker than their controls [47], but retinal water content and diffusion remain to be measured to determine whether or not this model displays retinal edema. Then, we examined the retinal outcome after a single intravitreal injection of a very potent and selective TRPV4 antagonist, GSK2193874 [39] in diabetic mice.

## Methods

### Reagents

The TRPV4 antagonist GSK2193874 and all other reagents were purchased from Sigma-Aldrich (St Louis, MO).

### Ethics statement

All experiments were approved by the Bioethics Committee of the Institute of Neurobiology at the National Autonomous University of Mexico (UNAM, protocol 74), and methods were carried out in accordance with the National Institutes of Health Guide for the Care and Use of Laboratory Animals, the ARVO Statement for the Use of Animals in Ophthalmic and Vision Research, and with authorization from the Institutional Animal and Care Use Committee.

### Animals

C57BL/6J mice of either sex (5–7 weeks old) were obtained from commercial suppliers, whereas *Trpv4*^-/-^ mice [48] were contributed by one of us (WL). Animals were fed *ad libitum* and reared in normal cyclic light conditions (12 h light: 12 h dark) with an ambient light level of approximately 400 lux.

Diabetes was induced with intraperitoneal injections of streptozotocin (60 mg/kg) once a day for five consecutive days [49]. Animals with glucose levels greater than 250 mg/dL after a 6-h fast [50] were used 4 weeks after diabetes induction. Nondiabetic groups received intraperitoneal injections of citrate buffer once a day for five consecutive days (controls). Body weight and glycemia were monitored weekly (S1 Fig).

In addition to the wild-type and *Trpv4*^-/-^ mice rendered or not diabetics, the study included 4 additional groups; control and diabetic mice that were randomized to receive an intravitreal injection with PBS or TRPV4 antagonist. The final injection volume was 0.5 μl. In both diabetic and nondiabetic mice, the right eye was injected with vehicle (PBS) and the left eye received GSK2193874 (17.3 pg, corresponding to 50 nM, as the estimated volume of mouse vitreous is 4.4 μl [51]). We previously demonstrated that PBS is an acceptable vehicle [8]. Based on a previous study showing that 24 h after intravitreal injection of TRPV4 channel inhibitors, RN1734 and GSK2193874, inhibition of the streptozotocin-induced BRB breakdown was observed [8], intravitreal injection was performed 24 hours before the end of the 4 weeks of diabetes to mimic a treatment scheme.

Mice were anesthetized intraperitoneally with ketamine (80 mg/kg) and xylazine (10 ml/kg) before intravitreal injections and MRI procedures. To maintain light conditions between all groups, and given that the magnet bore was dark, mice were manipulated under dim red light and *ex vivo* imaging was performed in dark-adapted mice (for at least 12 h).

### Ex vivo MRI procedures

Anesthetized mice were perfusion-fixed with 4% paraformaldehyde and gadolinium (2 μM) in PBS and stored at 4 °C [52]. Mice were decapitated after fixation. Samples were allowed to stabilize at room temperature (21 ± 1 °C) for 4 h before image acquisition. High-resolution anatomic and ADC data were acquired using a 7.0 T system (Bruker Pharmascan 70/16; Billerica, MA, USA), equipped with a gradient set with Gmax = 760 mT/m. To enhance signal-to-noise ratio, we used a two-channel Helium-cooled phased-array surface probe (Cryoprobe, Bruker), centered between both eyes. An off-resonance (i.e., B0) map was obtained and used to calculate high-order shim gradients through routines provided by the manufacturer (i.e., MapShim). Images were acquired using a spin-echo sequence with three-dimensional spatial encoding, TR = 1000 ms, TE = 21.55 ms, FOV = 12 x 9.04 x 2.4, matrix dimensions = 266 x 200 x 8, yielding a voxel resolution of 45 x 45 x 300 μm^3^, bandwidth = 30.864 kHz, NEX = 1. Slices were oriented perpendicular to the rostro-caudal axis, with imaging planes covering both eyes. Spectral fat suppression was performed using a preparation pulse with bandwidth = 1050 Hz. DWI were obtained with three orthogonal diffusion encoding directions with b = 1200 s/mm^2^, Δ = 8.5 ms, δ = 2.5 ms. In addition, two non-diffusion weighted volumes (i.e., b = 0 s/mm^2^) were obtained with identical parameters. Total data acquisition time was 1 h 40 min. Experiments were performed at room temperature controlled at 21 ± 1 °C. ADC maps were calculated as ADC = (ln(S/S_0_)) / -b, where S is the mean of the three DWI and S_0_ is the mean of the two non-diffusion weighted volumes.

### MRI data analysis

Images were analyzed using ITK-SNAP [53]. As discussed in [46], we inferred layer locations based on the retina´s well-defined laminar structure and clear anatomical landmarks like the vitreous-retina and neuroretina-choroid/retinal pigment epithelium borders. Total thickness and ADC values were quantified on each section every 1,000 μm from the edge of the optic nerve head to 30° in both nasal and temporal directions, and every 4,000 μm from the 30° radius to the ora serrata and averaged among the same groups. DWI images were used to assess retinal thickness, since these images allow for clear visualization of the retina as hyper-intense band.

Non-diffusion weighted images were used to check retinal structure after intravitreal injections. Of note, anatomical MRI revealed that some *Trpv4*^-/-^ eyes (2 out of 11) showed an enlarged lens capsule (S2 Fig).

### Histology

Mouse eyes were fixed for 3 hours at room temperature in 4% paraformaldehyde. Eyes were then cryopreserved at 4 °C for 1 h 30 in 10% and 20% sucrose, respectively, in 30% sucrose overnight, embedded in Tissue-Tek and frozen with liquid nitrogen. Cryostat was sectioned at 14 μm and mounted on slides for hematoxylin and eosin staining [8]. Retinal layer thickness was quantified, as previously described [8]. Histomorphometry was performed using the freely-available image analysis software ImageJ (V.1.36, National Institutes of Health). Only cropping of images was performed; there was no adjustment of brightness. Nuclei were detected using the ImageJ-Integration in KNIME Image Processing, in three fields (300 μm x 100 μm) distributed equally over the ONL, INL, and GCL from the whole retina at x40 magnifications in sections stained with hematoxylin/eosin. This process was repeated in three retina sections per condition. In these fields, the nucleus eccentricity, defined as the ratio of the distance between the centromere and the major axis length, was automatically determined, as well as the nucleus area. The mean internuclear distance, defined as the averaged distance between the center of the nucleus and all immediate neighbouring nuclei, was manually calculated in one field (90 μm x 50 μm), randomly chosen in each previously defined field. Values were averaged to determine the cell density, mean internuclear distance, nucleus area, and nucleus eccentricity in the four experimental groups.

### Statistical analysis

All results were replicated in three or more independent experiments. All data were reported as mean ± s.e.m. All data showed normal distribution and equal variance according to D’Agostino-Pearson omnibus and Levene’s tests, respectively. Comparisons between groups were determined by ANOVA (R Studio). Differences between means with *P* < 0.05 were considered statistically significant. We found no sex-related differences in any of the tested parameters (body weight, glycemia, retinal thickness, Evans blue concentration, and ADC), in none of the groups (STZ or *Trpv4*^-/-^) and therefore data were pooled.

## Results

### Group summary

*Trpv4*^-/-^ mice had body weight and glycemia comparable to those of wt mice (18.6 ± 0.6 g; *n* = 8 vs. 19.4 ± 1.1 g; *n* = 10 and 180.8 ± 7.5 mg/dl; *n* = 8 vs. 199.3 ± 7.8 mg/dl; *n* = 10, respectively; *P* > 0.05; S1 Fig). The 4-week streptozotocin treatment did not alter the body weight of wt and *Trpv4*^-/-^ mice (19.4 ± 0.4 g; *n* = 11 vs. 18.3 ± 0.5 g; *n* = 9; *P* > 0.05), but it induced hyperglycemia at similar levels (*P* > 0.05) in both groups (412.5 ± 22.9 mg/dl; *n* = 11 vs. 499.7 ± 4.7 mg/dl; *n* = 9, S1 Fig). Intravitreal injections of PBS or GSK2193874 did not modify nondiabetic and diabetic mouse body weight and glycemia (*P* > 0.05, data not shown).

### TRPV4 contributes to retinal structure and is necessary for BRB rupture and increased water diffusion in diabetic mouse retinas

**Fig 1:**
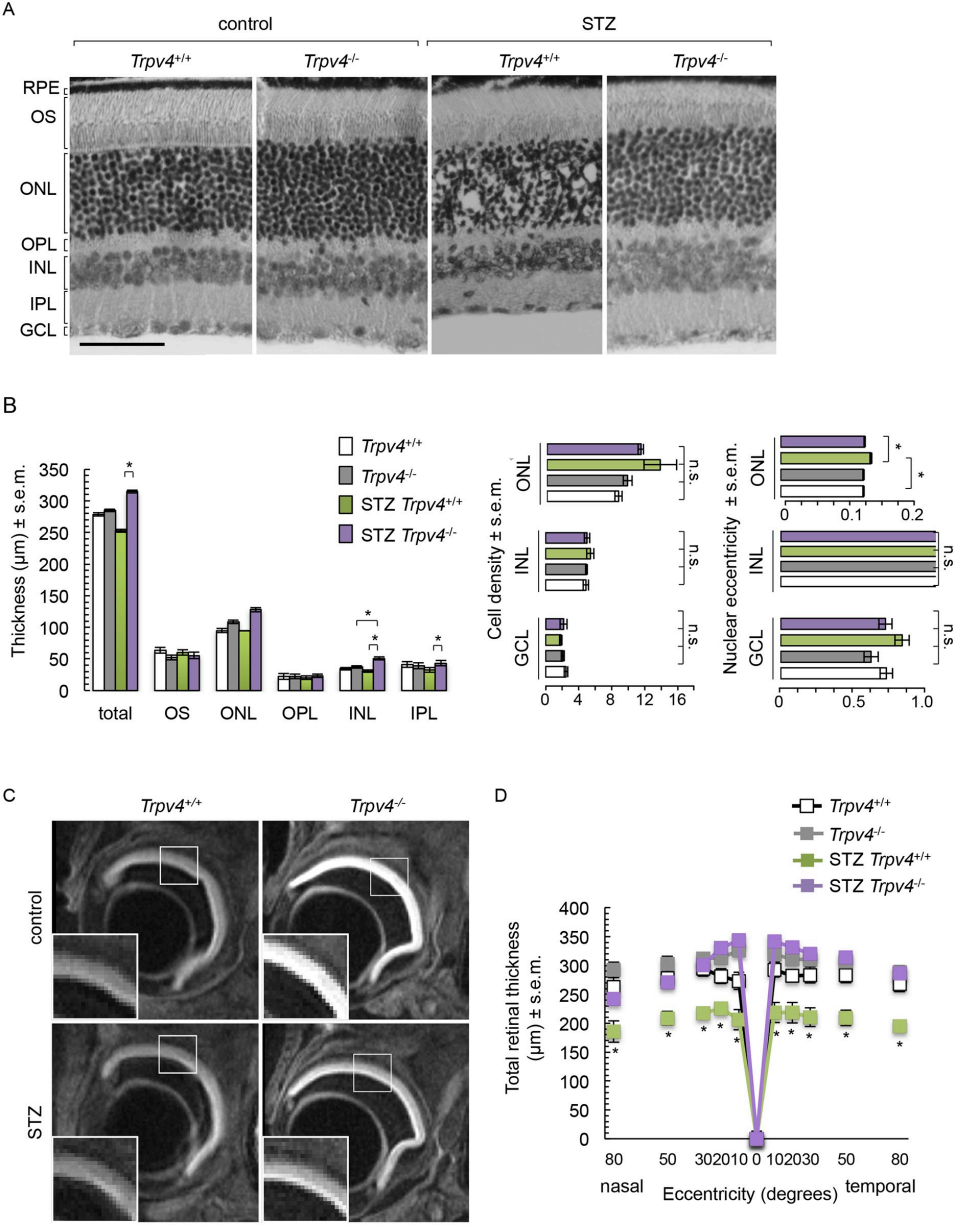
Retinal morphometry analysis in nondiabetic (control) and diabetic (STZ) wild-type (*Trpv4*^+/+^) and *Trpv4*^-/-^ mice. (**A**) Representative images of hematoxylin/eosin-stained retinas and (**B**) corresponding quantification of retinal thickness, cell density per mm^2^, and nuclear eccentricity. Retinal pigment epithelium (RPE), outer segments (OS), outer nuclear layer (ONL), outer limiting membrane (OLM), outer plexiform layer (OPL), inner nuclear layer (INL), inner plexiform layer (IPL), and ganglion cell layer (GCL). From each of six animals per group, three equally distributed sections (300 μm x 100 μm) throughout the entire retina were analyzed. Scale bar, 100 μm. (**C**) Representative DWI images with enlargements corresponding to white squares in control and STZ *Trpv4*^+/+^ and *Trpv4*^-/-^ mouse eyes. (**D**) Plots represent the thickness measurements (μm, mean ± s.e.m.; n = 8–11, N = 3) for retinas of control and STZ *Trpv4*^+/+^ (*white*) and *Trpv4*^-/-^ (*grey*) mice in DWI images. *, significant differences of P < 0.05. n.s., not significant.

The histology of *Trpv4*^-/-^ retinas appeared relatively similar to that of wild-type mice (Fig 1A). Cell bodies can be seen in both the outer and inner plexiform layers of *Trpv4*^-/-^ retinas (Fig 1A). Diabetic wt retinas showed a disorganized outer nuclear layer with misaligned photoreceptor nuclei and less round nuclei (Fig 1A). Additionally, cell bodies could be detected in the outer plexiform layer in diabetic wt retinas (Fig 1A). Cell density was similar in all groups and morphology of cell nuclei was comparable between the inner nuclear layer and ganglion cell layer of all groups (Fig 1B). The histology of diabetic *Trpv4*^-/-^ retinas appeared similar to that of nondiabetic *Trpv4*^-/-^ retinas, except for a thicker inner nuclear layer (Fig 1B). Compared to diabetic wt retinas, diabetic *Trpv4*^-/-^ retinas displayed an organized outer nuclear layer (Fig 1A) with photoreceptor nuclei morphology similar to nondiabetic controls (Fig 1B) and thicker inner retina (Fig 1B). Total and inner retina thickness was increased in diabetic *Trpv4*^-/-^ retinas (Fig 1B). We found no significant changes in retinal pigment epithelium (not shown) and outer retinal layer thickness (Fig 1B).

The retina was easily observable through DWI in all groups (Fig 1C). Quantification of DWI-retinal thickness showed that diabetic wt mice had thinner retinas compared to nondiabetic wt mice (Fig 1D). This effect was absent in diabetic *Trpv4*^-/-^ mice (Fig 1D). Retinal thickness in nondiabetic *Trpv4*^-/-^ mice was indistinguishable from that of nondiabetic wt mice (Fig 1D). These effects were not focal but observed throughout the retina (Fig 1D).

**Fig 2:**
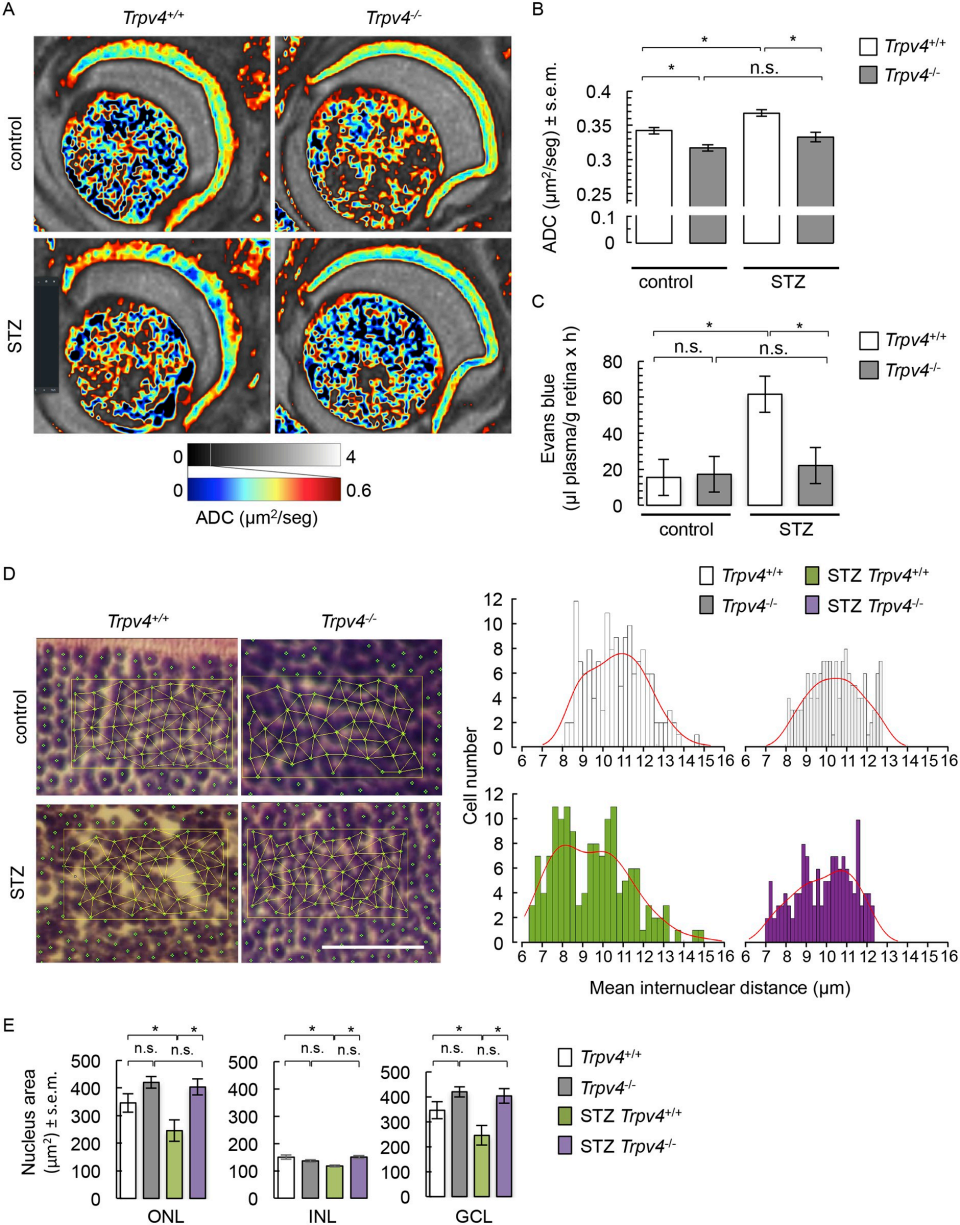
Water diffusion, BRB permeability, nuclear distance distribution, and nuclear area in retinas of control and STZ *Trpv4*^+/+^ and *Trpv4*^-/-^ mice. (**A**) Representative images of ADC maps in control and STZ *Trpv4*^+/+^ and *Trpv4*^-/-^ mouse eyes. ADC values between 0 and 0.6 μm^2^/s are displayed in color to enhance contrast, as the retina shows markedly reduced diffusion as compared to the vitreous. (**B**) Summary of ADC values in three separate groups of control and STZ *Trpv4*^+/+^ and *Trpv4*^-/-^ mice. (**C**) Evaluation of the Evans blue dye content in retinas from control and STZ *Trpv4*^+/+^ and *Trpv4*^-/-^ mice. For B and C, values are mean ± s.e.m. (n = 9–12 per group). (**D**) Representative fields (90 μm x 50 μm), randomly chosen in hematoxylin/eosin-stained retina images from control and STZ *Trpv4*^+/+^ and *Trpv4*^-/-^ mice, to illustrate how the distances between the center of the nucleus and all immediate neighbouring nuclei have been measured; summary of the distribution of mean internuclear distance evaluated in 3 fields per groups. Red line corresponds to data distribution. Scale bar, 50 μm. (**E**) Summary of the mean nuclear area in the ONL, INL, and GCL of control and STZ *Trpv4*^+/+^ and *Trpv4*^-/-^ mice (n = 3, N = 6). *, significant differences of P < 0.05; n.s., not significant.

Quantification of ADC maps (Fig 2A) showed an increase in retinal mean ADC values in diabetic wt mice (Fig 2B), an effect that was absent in diabetic *Trpv4*^-/-^ mice (Fig 2B). Nondiabetic *Trpv4*^-/-^ retinas displayed a decrease in mean ADC values compared with the nondiabetic wt group (Fig 2B). We also measured BRB breakdown using the Evans blue technique, given that in the streptozotocin mouse preclinical model of diabetes, BRB breakdown has been shown to occur as early as 2 weeks and up to 24 weeks post-streptozotocin treatment [54–58]. We confirmed the BRB breakdown in diabetic mice by showing that retinal accumulation of Evans blue-stained albumin tripled (Fig 2C). The streptozotocin-induced BRB breakdown was not observed in *Trpv4*^-/-^ mice (Fig 2C). As previously shown [14], lack of *Trpv4* did not modify the basal transport through the BRB (Fig 2C). To further support these observations, distance between neighboring nuclei was assessed. In the ONL of nondiabetic wt retinas, the mean internuclear distance followed a normal distribution, with cells being distant from their neighbors by 10.30 ± 0.01 μm (Fig 2D). In diabetic wt retinas, a bimodal distribution of intercellular distances can be distinguished with ~ half of the cells (47%) being distant from their neighbors by 7.87 ± 0.08 μm, and 53% of the cells being distant from their neighbors by 10.90 ± 0.13 μm (Fig 2D). Such inter-nuclear heterogeneity reflects the disarrangement of the ONL of STZ-treated *Trpv4*^*+/+*^ animals illustrated in Fig 1A. This effect was absent in diabetic *Trpv4*^-/-^ ONL, where cells are distant from their neighbors by 9.97 ± 0.12 μm (Fig 2D). The distance between photoreceptor cells was normal in nondiabetic *Trpv4*^-/-^ retinas (10.48 ± 0.11 μm; Fig 2D). The nuclear distance in the INL was similar in all groups (data not shown). In addition, the total surface of nuclei was reduced in all nuclear layers of diabetic wt retinas, effect that was absent in diabetic *Trpv4*^-/-^ mice (Fig 2E). The area of cell nuclei in nondiabetic *Trpv4*^-/-^ mice was similar to that of nondiabetic wt mice (Fig 1E).

### Effect of single intravitreal injection of TRPV4 antagonist GSK2193874 in diabetic mouse retina

**Fig 3:**
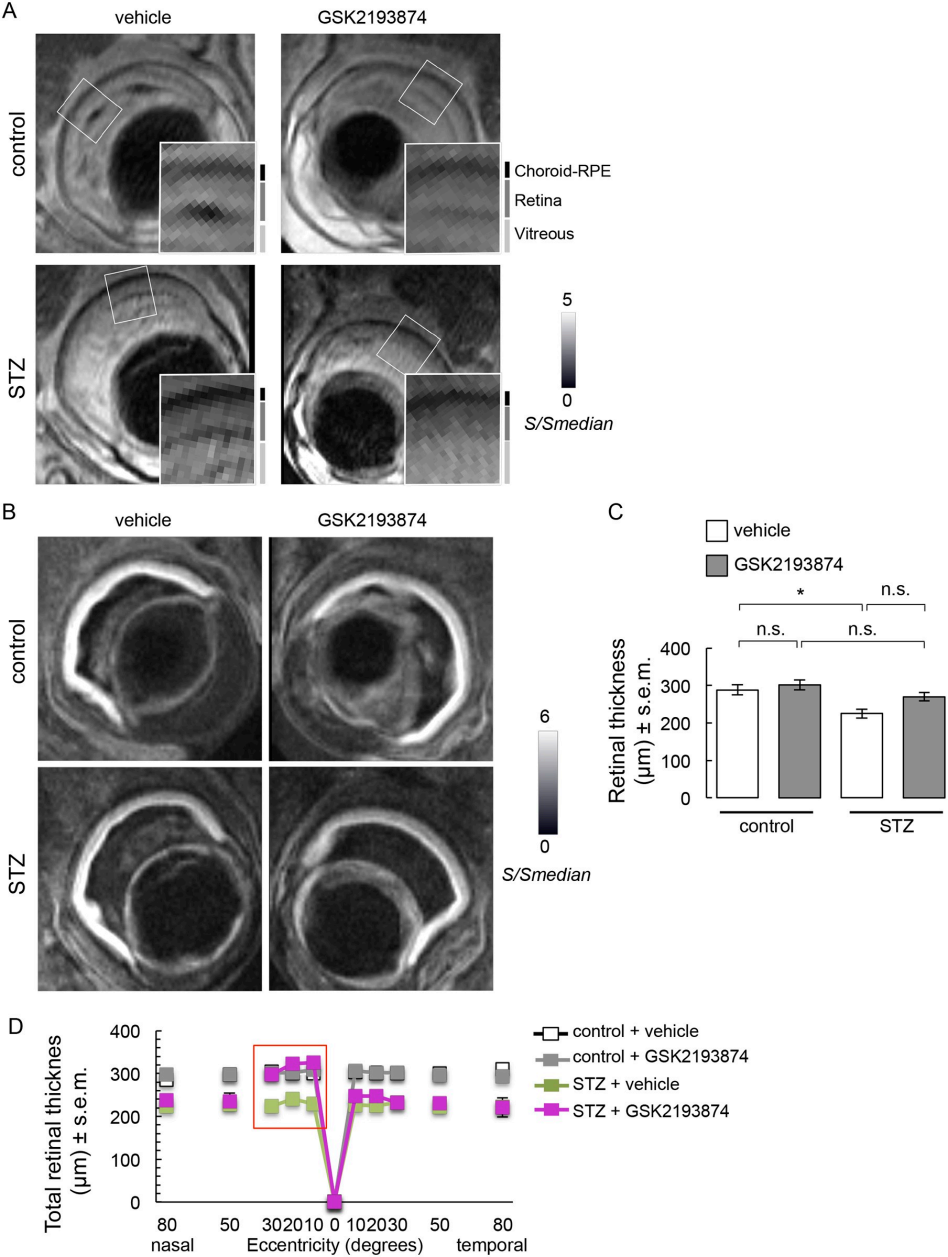
Retinal thickness assessment in control and STZ mice that received a single intravitreal injection of TRPV4 antagonist GSK2193874 on the left eye and vehicle (PBS) on the right eye, 24 h before the end of the 4-week STZ treatment. (**A**) Representative images of non-diffusion weighted images and enlargement corresponding to *white boxes*. Signal (*S*) is shown normalized with respect to the median signal intensity of the entire volume (*S*_*median*_). Approximate locations of vitreous, retina, and choroid-RPE complex are indicated in *vertical black and gray lines*. (**B**) Representative DWI showing the retina as a hyperintense band. (**C**) Summary of retinal thickness measurements from control and STZ mice treated with vehicle (*white*) and GSK2193874 (*grey*) in DWI images (n = 10–12, N = 4). *, significant differences of P < 0.05; n.s., not significant. (**D**) Corresponding plot of retinal-segment analysis (μm, mean ± s.e.m.). Vehicle-treated STZ retinas were significantly thinner than vehicle-treated control ones. GSK2193874-treated STZ retinas had similar thickness than vehicle-treated ones, except in the nasal central part of the retina (10–30 °, red box).

**Fig 4:**
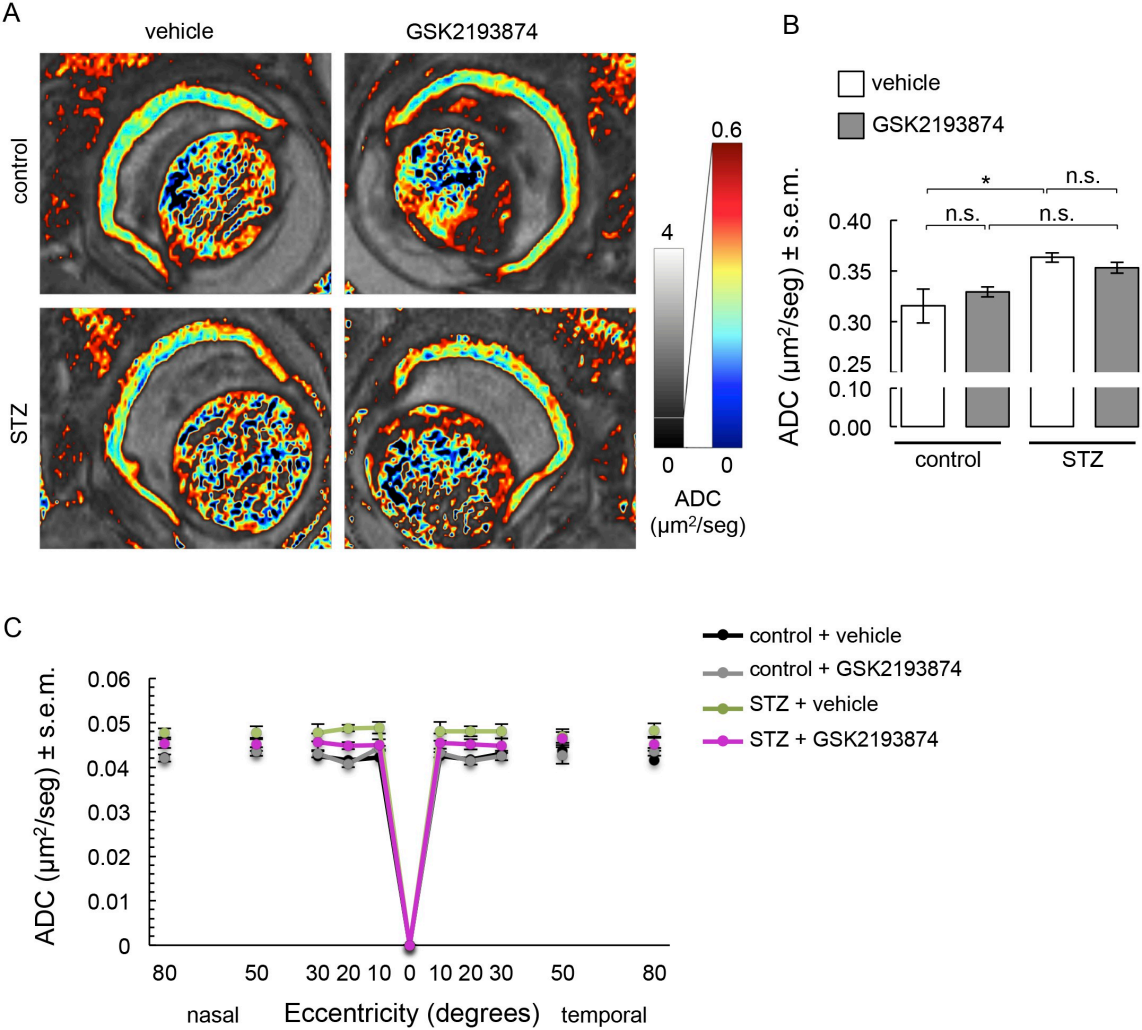
Water diffusion in control and STZ mice that received a single intravitreal injection of TRPV4 antagonist GSK2193874 on the left eye and vehicle (PBS) on the right eye, 24 hours before the end of the 4-week STZ treatment. (**A**) Representative images of ADC maps. (**B**) Summary of ADC and (**C**) corresponding retinal segment analysis in four separate groups. Error bars correspond to s.e.m. *, significant difference (P < 0.05); n.s., not significant. In C, vehicle-treated STZ retinas had a significantly higher ADC than that of vehicle-treated control retinas. The ADC of GSK2193874-treated STZ retinas was not different from that of vehicle-treated retinas and GSK2193874-treated control retinas.

Slight retinal/vitreous interface disruption at the intravitreal injection site was observed on the non-diffusion weighted images, but general structure was overall well conserved (Fig 3A). DWI data (Fig 3B) demonstrated that injection of either vehicle or GSK2193874 did not affect total retinal thickness when averaged throughout the retina (Fig 3C). Diabetic wt mice showed thinning of the retina if vehicle was administered (Fig 3C). This overall effect was not prevented by TRPV4 antagonist administration (Fig 3C). Further analysis showed that retinal thinning occurred throughout the entire retina of diabetic mice and GSK2193874 rescued retinas from streptozotocin-induced thinning in the nasal central part of the retina (Fig 3D). Using ADC maps (Fig 4A), we found a streptozotocin-induced increase in mean ADC values (Fig 4B) that was still present in the eyes of diabetic mice treated with GSK2193874 (Fig 4B). GSK2193874 did not modify mean ADC values in nondiabetic mice (Fig 4B). Fig 4C shows that mean ADC values increased throughout the entire retina of diabetic mice and that GSK2193874 did not block this increase.

## Discussion

This study examines whether endogenous TRPV4 regulates retinal hydro-mineral homeostasis under normal and diabetic conditions, using common metrics of retinal edema (morphology/thickness and BRB integrity) and DW-MRI. First, we observed that histological and MRI measurements of retinal thickness agreed in nondiabetic mice but not quite in diabetic mice. Retinal morphometry changes that include outer nuclear layer disorganization with fields with larger internuclear distances coincide with BRB breakdown and increased ADC in diabetic mouse retinas. These changes were not observed in the *Trpv4* knockout mouse, but they showed slower water diffusion. Furthermore, we found that a single administration of the selective TRPV4 antagonist GSK2193874 had no effect of retinal thickness and water diffusion in nondiabetic and diabetic retinas.

Our previous work showed that TRPV4 antagonists have significant therapeutic potential for the control of BRB breakdown in DME since they inhibit excessive permeation in the retina of the streptozotocin preclinical rat model of diabetes [8]. Measurement of BRB integrity is however insufficient to prove edema [59, 60]. MRI-based analysis of co-localized measures of retinal thickness, BRB breakdown, and water content and mobility has been established as an intergral approach to quantify retinal edema in diabetic rats [46]. Retinal water content was not assessed here because its quantification using MRI is based on assumptions.

The present data show the retina as a single MR-detected layer, as previously reported [61, 62]. Our MRI estimation of total retinal thickness in control wt mice (275.5 ± 6.1 μm) compares reasonably well with our own values using histology (278.7 ± 2.6 μm) and also with published values using histology, anatomical MRI and OCT from the same mouse strain (270.4 ± 2.3 μm [63], 237 ± 3 μm [64] and 222.3 ± 2.1 μm [65], respectively). This, however, does not stand true in diabetic mice, where MRI-based measurements showed retinal thinning and histological analysis did not. Additionally, a previous MRI study showed that retinal thickness was similar between control and diabetic male C57BL/6 [47]. In addition to possible variations between C57BL/6 mouse sub-strains, some critical differences (e.g. 1 month vs. 3 months of diabetes; *ex vivo* vs. *in vivo* imaging conditions; total vs. central retina, from current and [47] studies) may account for this inconsistency. The discrepancy between our MRI and histological values may also be attributed to differences in the dehydration process of eyes that were dissected prior PFA fixation in histology procedures, but not in MRI. Furthermore, histological examination has produced inconsistent results with thicknesses that are unchanged [43, 66] or reduced [67] at 4–5 weeks after onset of diabetes. At longer periods of diabetes, retinal thinning [43, 68–73] or thickening [47, 74] has been observed. Retinal thinning concurs with cell loss [43, 75] and not water loss [46] in diverse rodent models of short-term diabetes (< 4 months). We did not look for cell death but found similar cell density between groups. Diffuse central retinal edema causes retinal thickening in rats [46]. Current evidence, obtained from the whole retina, shows that indicators of retinal edema, i.e. BRB breakdown and increased water diffusion, do not necessarily associate with retinal thickening, if they match morphometric changes compatible with retinal edema. Indeed, our data show a binomial distribution of internuclear distances and smaller nuclei in the outer nuclear layer of diabetic wt retinas that mirrors the large pools devoid of nuclei in the ONL of diabetic wt animals illustrated in Fig 1A. These large inter-nuclear spaces may allow for increased water diffusion. Consistent with this interpretation, increased ADC is not homogenous in the whole retina section. Histological examination validates diffusion MRI.

Water diffusion was assessed in fixed tissue. *Ex vivo* imaging provides high resolution images but there are major differences between *ex vivo* and *in vivo* DWI. Temperature variations modulate diffusitivity. Because room temperature was kept at 21 °C in our experiments, overall diffusivity is reduced compared to *in vivo* measurements. Furthermore, under *ex vivo* conditions, there is no blood flow. This can lead to differences in overall water diffusivity, as the capillaries are filled with freely-diffusing water in the *ex vivo* condition. Conversely, blood flow in the living animal makes signal practically non-observable in DWI using long diffusion times). However, vasodilation has not been reported in retinas of diabetic animals [76–78] or of patients with early stages of diabetic retinopathy [79, 80]. Our data therefore indicate that diabetic C57BL/6 mice develop retinal edema.

Retinal water content remains to be estimated in diabetic C57BL/6 mice to undoubtedly conclude that this model displays retinal edema. Edema can be cytotoxic (intracellular) or vasogenic (extracellular); both occur in diabetic retinopathy [81]. Supernormal ADCs are usually found in models of BRB breakdown presumably because free water contained in leaking fluid accumulates into the extracellular space [45, 46, 82]. In contrast, the intra-cellular environment is thought to restrict water diffusion, rendering low ADC values in the case of cytotoxic edema [83]. Our results likely identify vasogenic (or interstitial) edema in diabetic mouse retinas because they show increased water diffusion and loss of BRB integrity. Nevertheless, we cannot specifically attribute the large irregular spaces between photoreceptor nuclei to increased extracellular space or cell swelling.

The reason for subnormal ADC values in *Trpv4*^-/-^ retinas is somewhat unclear. Intracellular swelling may cause reduced water mobility. In this sense, TRPV4 mediates regulatory volume decrease—i.e., decrease in swelling in the presence of sustained hypotonic stress [6, 84]—by transducing increases in Müller cell [7] and retinal neuron [85] volumes into Ca^2+^ signals. However, TRPV4 antagonism suppresses cell volume expansion [86]. Cell density in *Trpv4*^-/-^ retinas was comparable to that of wild-types. Subnormal ADC can also relate to vasoconstriction and literature supports that TRPV4 activation causes vasodilation [17, 87]. Additionally, together with aquaporin-4 and Kir4.1 channels, TRPV4 forms a functional complex that maintains the steady-state ‘osmo-tensile’ homeostasis at Müller glial endfeet [7, 88]. More work is needed to test whether TRPV4 contributes to retinal hydro-mineral homeostasis through an extended vascular-glial-RPE-ganglion cell network, as shown in other sensory systems [48, 89, 90].

We found that TRPV4 deletion abolishes retinal edema in streptozotocin-treated mice, demonstrating that TRPV4 contributes to the formation of retinal edema in diabetic conditions. Our finding coincides with the promoting effect of TRPV4 agonism on barrier permeability [12, 13, 15, 16, 18, 34]. That streptozotocin-treated mice likely present vasogenic edema (present data) and that this latter associates with inner BRB breakdown [5], suggests that TRPV4 acts on the inner BRB components. In apparent contrast with our data, expression levels of TRPV4 decrease in retinal microvascular vessels from streptozotocin-induced diabetic rats [8]. Nevertheless, TRPV4 is still functional in the diabetic retina [8, 20]. The activity of remaining TRPV4 may be excessive since the amount and nature of TRPV4 endogenous agonists and the levels of glycosylation, free intracellular Ca^2+^, and membrane cholesterol, all of which regulate TRPV4 activity, are altered in diabetic milieu [91–94]. As previously proposed [8], reduced TRPV4 expression may be a compensatory effect in diabetes.

Our data further suggest that TRPV4 participates in the effect of diabetes on retinal fluid accumulation. Based on the facts that (i) VEGF-mediated angiogenesis and inflammation act interdependently in the development of DME [81], (ii) TRPV4 agonism associates with increased expression of VEGF [95] and amplifies inflammatory cascades [29–32, 34], and (iii) TRPV4 antagonism and anti-angiogenic molecules synergize by activating complementary pathways to counteract the diabetes-like effects on outer BRB permeability [8], it is possible that TRPV4 blockade targets both the vasogenic and inflammatory pathways in diabetic retina [96]. *Trpv4*^-/-^ mice treated with streptozotocin are hyperglycemic, in contrast to littermates subjected to a high-fat diet [97], suggesting that the protective effect of TRPV4 inhibition against retinal edema is independent from its protective effect against insulin resistance. The exact underlying mechanism needs to be clarified, but the fact that TRPV4 deletion prevents retinal edema formation associated with diabetes argues in favor of TRPV4 as an etiological agent for DME.

GSK2193874 has no reported systemic side effects [39, 98] and we confirm herein that short-term inhibition of TRPV4 does not alter BRB permeability [8]. Previous findings showed that TRPV4 blockade preserves the lung [39] and brain from vasogenic edema [40]. We found that a single injection of GSK2193874 does not revert streptozotocin-induced retinal edema in mice but locally mitigates retinal thinning. The effect appears limited to a peripheral region deviated towards the temporal side, corresponding to the injection site. We consider that a scheme of repeated injections of GSK2193874, as well as the generation of ophtalmological presentations for TRPV4 antagonists may improve this current limitation. While delivery of GSK2193874 has some injection-related side effects [99, 100], they are uncommon and the intravitreal route ensures that the drug reaches the target site. In this line, we emphasize that the availability of potent and selective drugs to inhibit TRPV4 is very important, not only because all 26 members of the mammalian TRP family are present in the retina [101], but also to favor the curative use of GSK2193874. Because type 1 and type 2 models of diabetes exhibit similar early signs of diabetic retinopathy [80], our findings may contribute to treatment development for DME arising from both type 1 and type 2 diabetes. This is of importance considering that more than 90% of diagnosed patients suffer from a type-2 diabetes phenotype. Further studies using *in vivo* DWI and OCT are also warranted to help determine responses to potential treatment.

We conclude that TRPV4 contributes to the cascade of events that involve the breaking of BRB, which is the stage prior to DME onset. Further experiments are needed to test whether selective inhibition of TRPV4 by GSK2193874, other selective inhibitors, and loss-of-function strategies have disease-modifying potential for patients with DME. The role of TRPV4 may also extend to other types of retinal edema.

## Supporting information

S1 Fig Follow-up of body weight and glucemia in control and STZ *Trpv4*^+/+^ and *Trpv4*^-/-^ mice. *, significant difference (P < 0.05); **, significant difference (P < 0.025); n.s., not significant.
(TIF)

S2 Fig Anatomical MRI illustrating that some *Trpv4*^-/-^ mice (two out of eleven) showed enlarged lens capsule (delimited by *white dashed circles*) compared with *Trpv4*^+/+^ mice. (TIF)
