# Supplementary figures and images for: TRPV4 inhibition prevents increased water diffusion and blood-retina barrier breakdown in the retina of streptozotocin-induced diabetic mice
Source: PLoS One. 2019 May 2;14(5):e0212158. doi: 10.1371/journal.pone.0212158 (PMC6497373; doi:10.1371/journal.pone.0212158)

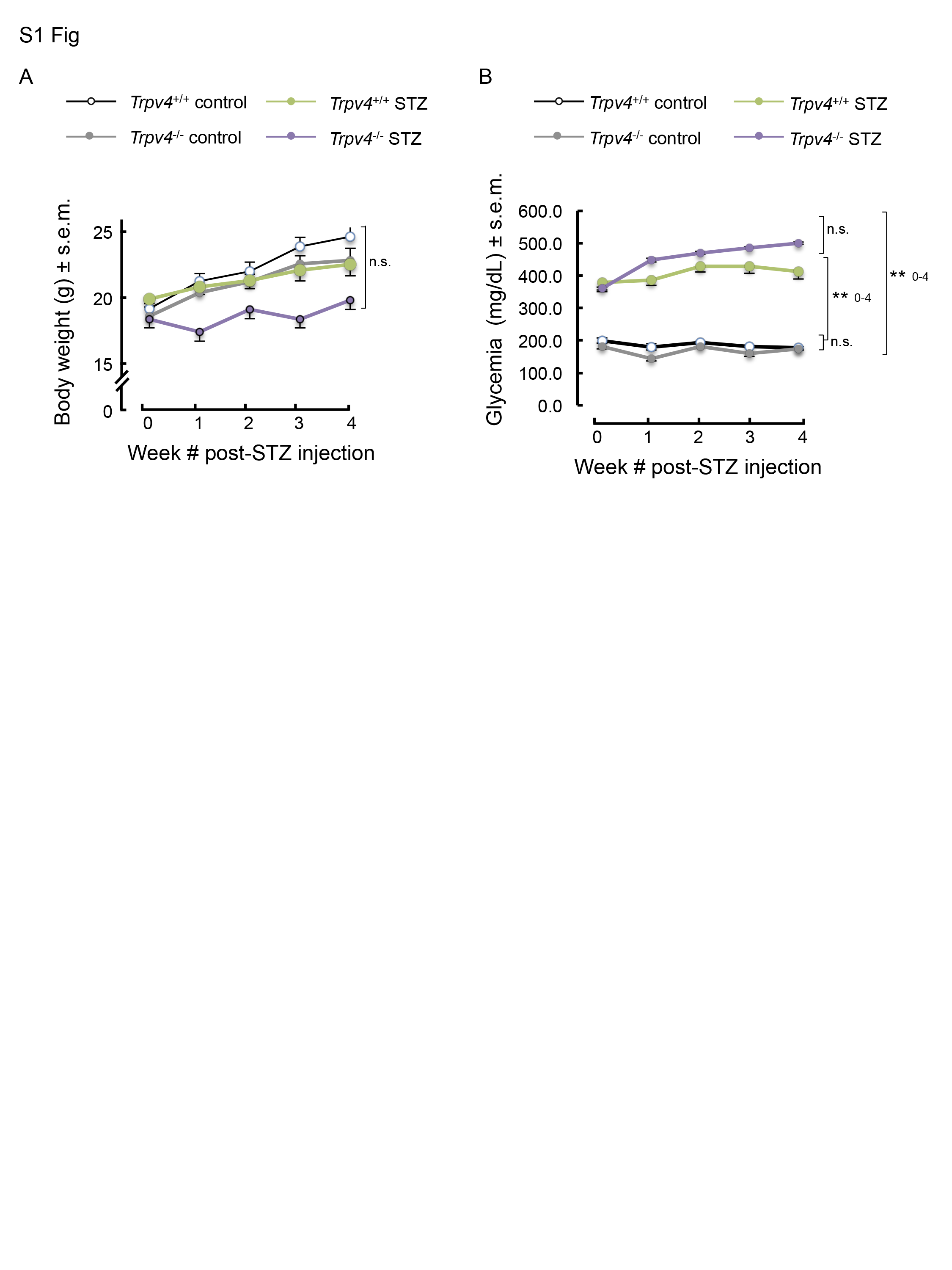

Supplement: S1 Fig — *, significant difference (P < 0.05); **, significant difference (P < 0.025); n.s., not significant. (TIF) [file pone.0212158.s001.tif]

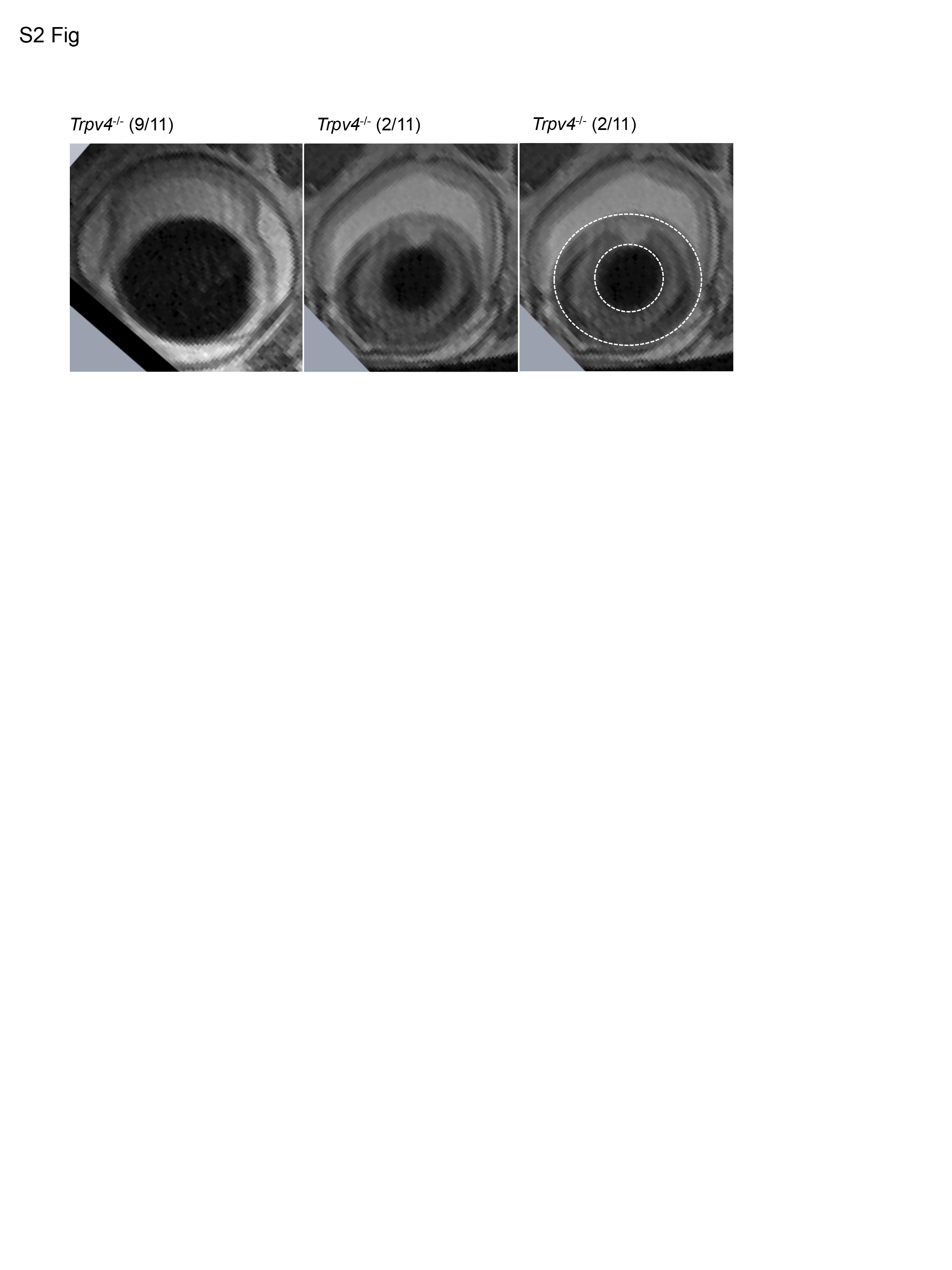

Supplement: S2 Fig — (TIF) [file pone.0212158.s002.tif]
